# The measurement of immunosuppressive drugs bymass spectrometry and immunoassay in a SouthAfrican transplant setting

**DOI:** 10.1016/j.plabm.2024.e00440

**Published:** 2024-11-06

**Authors:** Amy Strydom, Doreen Jacob, Taryn Pillay, Refeletse Malahlela, Sean Currin

**Affiliations:** Department of Chemical Pathology, National Health Laboratory Service and University of the Witwatersrand, Johannesburg, South Africa

**Keywords:** Immunoassay, Immunosuppressants, Mass spectrometry, Therapeutic drug monitoring

## Abstract

**Objectives:**

Liquid chromatography tandem mass spectrometry (LC-MS/MS) is the gold standard for measurement of immunosuppressive drugs (ISDs), but is technically demanding and less accessible in resource-limited countries. Immunoassays can also measure ISD concentrations, but may be limited by cross-reactivity. We evaluated the performance of the Roche electrochemiluminescence immunoassay (ECLIA) for cyclosporine, everolimus and sirolimus against LC-MS/MS in an African population for the first time.

**Methods:**

Bias for ECLIA was estimated by comparing ECLIA-measured ISD concentrations to those obtained by LC-MS/MS in 42, 43 and 47 patient samples for cyclosporine, everolimus and sirolimus, respectively. Precision was assessed by performing replicate measurements of quality control materials.

**Results:**

Deming regression analysis for all ISDs showed strong correlation between ECLIA and LC-MS/MS with a Pearson's r of >0.94. The slopes for cyclosporine, everolimus and sirolimus were 0.94 [95 % CI: 0.87–1.03], 1.35 [95 % CI: 1.23–1.44] and 0.96 [95 % CI: 0.85–1.15] with y-intercepts of 31.60 μg/L [95 % CI: 2.02–57.63], 0.23 μg/L [95 % CI: 0.21 – 0.72] and 2.61 μg/L [95 % CI: 1.30–3.56], respectively. Difference plots showed a median bias of 2.07 % [95 % CI: 1.42 – 6.99 %], 41.2 % [95 % CI: 34.9–51.8 %] and 34.9 % [95 % CI: 28.4–47.3 %] for cyclosporine, everolimus and sirolimus, respectively.

**Conclusions:**

The cyclosporine ECLIA yielded results comparable to LC-MS/MS while poorly comparable results were obtained for everolimus and sirolimus, which may be explained by ISD metabolite cross-reactivity, amongst other factors. The poor comparability, although not unique, is noteworthy and the clinical consequences of these differences require further investigation.

## 1 Introduction

Immunosuppression, achieved with the use of immunosuppressive drugs (ISDs), plays a vital role in preventing solid organ rejection post-transplantation [1]. In the 1980s, the introduction of cyclosporine revolutionised post-transplant care, with reported improvements in graft survival rates of more than 80 % compared to older drug regimens [2]. Further developments, including the introduction of newer drugs with fewer adverse effects, such as tacrolimus, everolimus and sirolimus has allowed for improved one-year graft survival of more than 90 % in most transplant centres [[3], [4], [5]]. In the South African tertiary public hospital setting, despite limited resources, reported 1-year graft survival rates for adult patients vary between 89.4 % and 91.7 % for renal transplants [6,7] and 72 %–81 % for liver transplants [8,9]. While the reasons for graft rejection are multi-factorial, some of the major modifiable risk factors leading to decreased survival include non-adherence and inadequate circulating concentrations of ISDs [10,11].

Due to risk of toxicity, as well as the risk of organ rejection from sub-therapeutic ISD concentrations, numerous groups have published guidelines and consensus reports mandating therapeutic drug monitoring (TDM) for patients on ISDs (ISD-TDM) [[11], [12], [13], [14]]. The gold standard method for measurement of ISDs is liquid-chromatography tandem mass spectrometry (LC-MS/MS) [14,15]. This method provides good analytical sensitivity and has been shown to be highly specific for the measurement of ISDs [16]. LC-MS/MS is, however, costly to run, requires highly skilled staff, has complex method validation, limited automation and little manufacturer support [15,16]. Immunoassay methods are also used for ISD-TDM and address some of the limitations of LC-MS/MS by being easily implementable, having less stringent skill requirements from staff, and the potential for shorter turn-around times as part of a 24/7 laboratory service [17]. The main limitation of the use of immunoassay methods is decreased specificity due to cross-reactivity [18].

Currently, there are no published studies assessing immunoassays for ISD-TDM in an African population [[19], [20], [21], [22], [23], [24], [25], [26], [27], [28], [29], [30], [31], [32], [33], [34], [35]]. Drug metabolism of ISDs and the drug metabolites formed may vary depending on the pharmacogenomic profile of a population [[36], [37], [38]]. The presence of various single nucleotide polymorphisms (SNPs) in the cytochrome P450 (CYP450) system are known to cause clinically relevant alterations in drug metabolism in different populations [39]. African populations, known to have greater genetic variation than other populations, have been shown to house CYP450 SNPs of serious clinical consequence [40,41]. SNPs in the genes encoding for CYP3A4 and CYP3A5, the major enzymes involved in the metabolism of cyclosporine, everolimus and sirolimus, have been identified in the South African population, but the clinical implications of these findings are yet to be explored [41,42]. A South African study that investigated the presence of various CYP3A5 SNPs in 43 transplant patients reported differences in ISD dosing requirements based on the presence of these SNPs [43].

The implications of variable pharmacokinetics and the inherent cross-reactivity of drug metabolites for immunoassay-based measurements make the verification of an immunoassay's analytical performance within that patient population a necessity. In this study, the first to be performed in an African population, the performance of the Roche Electrochemiluminescence Immunoassay (ECLIA) for cyclosporine, everolimus and sirolimus was evaluated against the gold standard LC-MS/MS method through a method comparison experiment to determine its suitability in an African population.

## 2 Methods

### 2.1 Setting

The study took place at the University of Witwatersrand and National Health Laboratory Service (NHLS) Laboratory in Johannesburg, South Africa. The laboratory services the Charlotte Maxeke Johannesburg Academic Hospital (CMJAH) and surrounding areas. Ethical approval was obtained for the study from the Health Research Ethics Committee of the University of the Witwatersrand (M230642).

### 2.2 Sample collection

For the method comparison, a total of 50 remnant whole blood EDTA samples, with concentrations of ISDs spanning the analytical range of the immunoassay and medical decision limits, were collected for each drug over 3 months. Each whole blood sample was aliquoted into a 2.0 mL Provetta cryo-vial and stored at −80 °C for less than 6 months (as per manufacturer recommendation and stability studies [[44], [45], [46]]) until analysis. The frozen samples were thawed for 45 min and gently mixed to ensure homogeneity. Each whole blood sample was then aliquoted into two Eppendorf tubes for repeat analysis by LC-MS/MS, followed by ECLIA measurement within 2 h of the LC-MS/MS analysis. For the precision study, Roche Diagnostics quality control (QC) materials (PreciControl® ISD and PreciControl® Everolimus) were used.

### 2.3 Method comparison

#### 2.3.1 Analysis by ECLIA

For the measurement of cyclosporine, everolimus and sirolimus, the samples require pre-treatment using 300 μL of Elecsys® ISD sample pre-treatment solution, followed by centrifugation for 4 min at 10 000 g. Calibration was performed using Elecsys® CalSet calibrators and QC was within acceptable limits. Analysis of 150 μL of the supranatant of these samples was performed using the Roche Cobas e602 module as per the manufacturer's instructions. Measurement by this assay is based on a competitive principle between the drugs and a ruthenium-labelled analogue. After a voltage is applied, an electrochemiluminescent signal is used to determine the drug concentration [44].

All samples were analysed within both the uncapped and capped stability period of 30 min and 4 h respectively, after the pre-treatment step as per manufacturer instructions.

#### 2.3.2 Analysis by LC-MS/MS

Analysis by LC-MS/MS took place in an ISO 15189 accredited laboratory that participates in the Randox International Quality Assessment (RIQAS) external quality assessment scheme for ISDs. Analysis of cyclosporine, everolimus and sirolimus by LC-MS/MS was performed on an ExionLC™ AC Series coupled to a Sciex® QTRAP 4500 tandem mass spectrometer. Chromsystems® calibrator standards were used to perform a 6-point calibration to allow for quantitation of each drug. Analysis of QC using Whole Blood Chromsystems MassCheck® control materials (levels 1–4) was within acceptable limits.

Each aliquot of sample was processed with the addition of Chromsystems® Immunosuppressant deuterated internal standards (Cyclo-d12, Evero-d4, Siro-d3), cell lysis and protein precipitation using a haemolysate (Zinc Sulphate) and acetonitrile, followed by centrifugation at 10 000 g for 5 min. The pre-treated samples were separated by gradient reverse phase chromatography using a Waters® ACQUITY UPLC BEH-phenyl column - mobile phase A (5 mM ammonium acetate and 0.1 % formic acid in water) and mobile phase B (0.1 % formic acid in acetonitrile). Electrospray ionization (ESI) was operated in positive mode. The tandem mass spectrometer was operated in multiple reaction monitoring (MRM) for the detection and quantitation of each drug using precursor and product ion *m*/*z* mass transitions. Quantitation of each drug was performed using QuanLynx software and a six-point standard curve.

### 2.4 Precision study

For 4 days, two levels of QC materials were analysed 3 times per day for each drug for the ECLIA method on the Roche Cobas® e603 instrument. For each analysis, a separate 300 μL aliquot of reconstituted QC material was subjected to the same pre-treatment procedure as the patient samples using Elecsys® ISD sample pre-treatment solution as described previously.

### 2.5 Statistical analysis

Statistical analysis was performed using R statistical software [47]. For the method comparison, results of the LC-MS/MS analysis in which there was a >15 % difference between the initial LC-MS/MS analysis and the single repeat LC-MS/MS analysis were excluded from the data set, which left 42, 43 and 47 data points for the method comparisons of cyclosporine, everolimus and sirolimus, respectively. The single repeat LC-MS/MS result was used for the method comparison. Deming regression analysis was performed for each drug with confidence intervals (CI) determined using the Bootstrap (quantile) method. Difference plots were constructed to assess bias. The median bias and its confidence intervals were calculated using the Wilcoxon signed rank test. The two methods were further evaluated by determining how many samples would be misclassified as sub-therapeutic, therapeutic or supra-therapeutic when comparing LC-MS/MS to ECLIA. The McNemar test was used to assess the significance of discordant classification between the two methods.

The within-run and within-laboratory variability estimates were calculated using published precision estimation formulae [48]. The laboratory's analytical coefficient of variation (CVa) for the LC-MS/MS method and the CVa data from the precision study were used to calculate the error variance ratios (δ) for the Deming regressions.

## 3 Results

Information about the collected samples is summarised in Table 1.

**Table 1:** Demographic data.

**Cyclosporine**	
Age	39 [31–59]
Sex (% male)	55 %
LC-MS/MS	333.4 μg/L [150.4–710.6]
ECLIA	342.6 μg/L [162.5–714.2]
**Everolimus**	
Age	35 [20–51]
Sex (% male)	60 %
LC-MS/MS	5.1 μg/L [2.5–6.9]
ECLIA	6.8 μg/L [3.6–9.2]
**Sirolimus**	
Age	44 [39.5–59.5]
Sex (% male)	57 %
LC-MS/MS	6.8 μg/L [5.4–8.9]
ECLIA	9.5 μg/L [7.5–10.7]

All data are presented as median and inter-quartile range (IQR) or as percentages (%).

### 3.1 Method comparison and estimation of bias

**Fig. 1:**
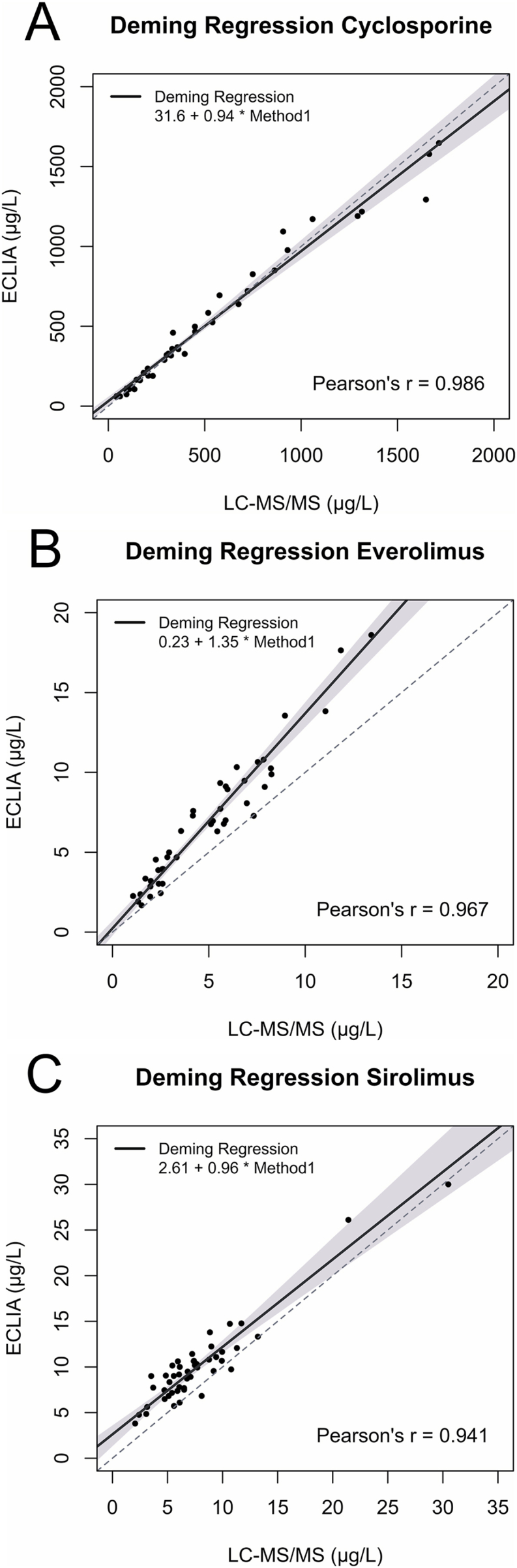
Deming regression analyses for (A) cyclosporine, (B) everolimus, (C) sirolimus. The dashed line is the identity (1:1) line and the grey shaded area represents the 95 % confidence interval for each drug.

As illustrated in Fig. 1, Deming regression analysis (δ = 0.46) for cyclosporine yielded a slope of 0.94 [95 % CI: 0.87–1.03], a y-intercept of 31.60 μg/L [95 % CI: 2.02–57.63] and a Pearson's r value of 0.986 [95 % CI: 0.973–0.992]. For everolimus, Deming regression analysis (δ = 0.76) yielded a slope of 1.35 [95 % CI: 1.23–1.44], a y-intercept of 0.23 μg/L [95 % CI: 0.21 – 0.72] and a Pearson's r value of 0.967 [95 % CI: 0.939–0.982]. For sirolimus, Deming regression analysis (δ = 0.51) yielded a slope of 0.96 [95 % CI: 0.85–1.15], a y-intercept of 2.61 μg/L [95 % CI: 1.30–3.56] and a Pearson's r value of 0.941 [95 % CI: 0.896–0.967].

**Fig. 2:**
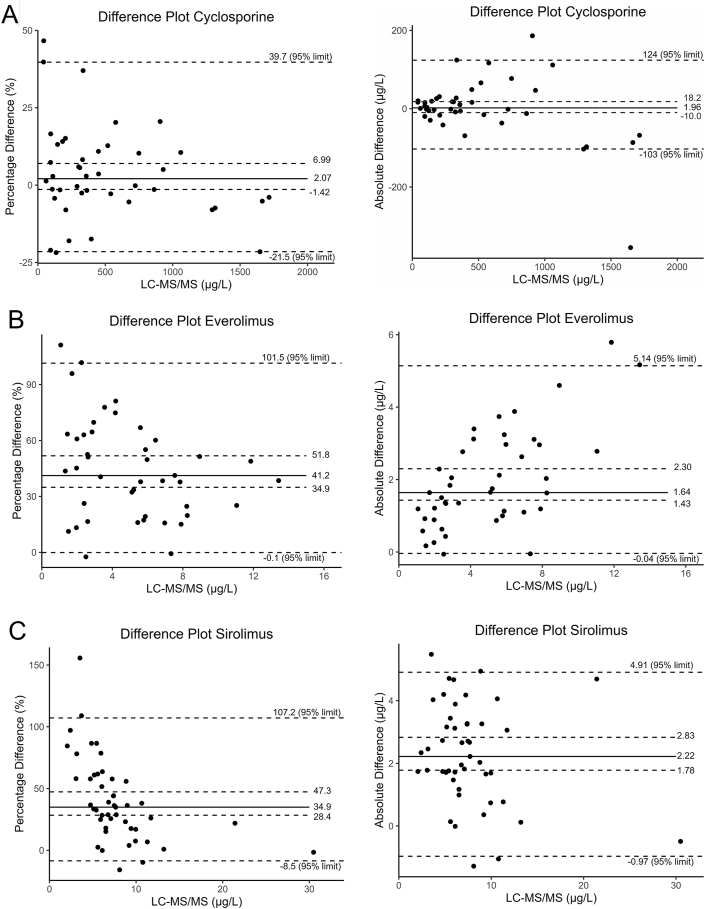
Difference plots (% difference and absolute difference for (A) cyclosporine, (B) everolimus and (C) sirolimus. The black solid line indicates the median value. The inner dashed lines indicate the confidence interval for the median difference. The outer dashed lines indicate the 95 % limits of the percentage and absolute difference data sets for each drug.

Difference plots (Fig. 2) for the cyclosporine method comparison revealed a median absolute difference of 1.96 μg/L [95 % CI: 10.0 μg/L - 18.2 μg/L], corresponding to a median percentage difference of 2.07 % [95 % CI: 1.42 %–6.99 %]. For everolimus, difference plots showed a median absolute difference of 1.64 μg/L [95 % CI: 1.43 μg/L - 2.30 μg/L] and a median percentage difference of 41.2 % [95 % CI: 34.9 %–51.8 %], while sirolimus showed a median absolute difference of 2.22 μg/L [95 % CI: 1.78 μg/L - 2.83 μg/L] and a median percentage difference of 34.9 % [95 % CI: 28.4 %–47.3 %].

There was a total of 12 misclassifications (27.9 %, p = 0.001) for the ECLIA method compared to LC-MS/MS for everolimus (Table 2) and 9 misclassifications (19.2 %, p = 0.008) for sirolimus, while no samples were misclassified for cyclosporine. For everolimus and sirolimus, the ECLIA method classified more samples as supra-therapeutic and fewer as sub-therapeutic when compared to LC-MS/MS.

**Table 2:** Classification of samples per therapeutic category for ECLIA and LC-MS/MS.

Drug	Method	Sub-therapeutic^a^	Therapeutic^a^	Supra-therapeutic^a^	Total disagreement (ECLIA vs LC-MS/MS)
Cyclosporine	ECLIA	8	17	17	0 (0 %)
	LC-MS/MS	8	17	17	
Everolimus	ECLIA	12	17	14	12 (27.9 %)^b^
	LC-MS/MS	17	19	7	
Sirolimus	ECLIA	1	39	7	9 (19.2 %)^b^
	LC-MS/MS	6	38	3	

a Based on therapeutic steady-state trough concentration range of 100–400 μg/L for cyclosporine, 3–8 μg/L for everolimus, 4–12 μg/L for sirolimus in keeping with local recommendations [13,44].

b p-value <0.05 using McNemar's test.

### 3.2 Precision

The within-run and within-laboratory precision estimates (Table 3) for cyclosporine, everolimus and sirolimus were <10 % at both clinically relevant concentrations of QC material.

**Table 3:** Within-run and within-laboratory precision estimates (CVa) of cyclosporine, everolimus and sirolimus by ECLIA.

Drug and QC Level		Within-run Precision	Within-lab Precision
Cyclosporine	84 μg/L	3.02 %	2.76 %
	272 μg/L	5.98 %	6.40 %
Everolimus	2.60 μg/L	5.32 %	5.71 %
	10.10 μg/L	9.60 %	9.93 %
Sirolimus	2.74 μg/L	5.98 %	6.68 %
	9.13 μg/L	4.65 %	3.83 %

## 4 Discussion

This study, based in South Africa, is the first to evaluate the analytical performance of an immunoassay for TDM-ISD in an African population through a method comparison between the Roche Elecsys® ECLIA method and the gold standard for TDM-ISD, LC-MS/MS. We found that the Roche Elecsys® ECLIA immunoassay and LC-MS/MS methodologies were comparable for cyclosporine, but not for everolimus and sirolimus.

There are a number of published method comparison studies for ISD immunoassays [[19], [20], [21], [22], [23], [24], [25], [26], [27], [28], [29], [30], [31], [32], [33], [34], [35]]. In general, literature on the performance of cyclosporine, everolimus and sirolimus immunoassays is inconsistent [[19], [20], [21], [22], [23], [24], [25], [26], [27], [28], [29], [30], [31], [32], [33], [34], [35],^49^] compared to tacrolimus [[19], [20], [21], [22], [23], [24], [25]]. Cyclosporine immunoassays, previously plagued by extensive cross-reactivity of drug metabolites [49], have shown improved analytical performance since the introduction of assays utilising monoclonal antibodies [[25], [26], [27], [28]]. For everolimus measurement, some studies reported adequate performance [25,32], while others noted biased and inconsistent results with the potential for clinical misinterpretation [30,31]. Similarly, method comparison studies for sirolimus immunoassays reported varied bias estimates, but overall were assessed as being fit for use [25,[32], [33], [34], [35]]. Due to cost constraints, we omitted the evaluation of tacrolimus from our study, as there is an extensive body of literature reporting acceptable analytical performance by immunoassay for this drug [[19], [20], [21], [22], [23], [24], [25]].

Our study demonstrates strong correlation (Pearson's r > 0.94) between the Roche ECLIA method and LC-MS/MS for cyclosporine, everolimus and sirolimus. Cyclosporine demonstrated a clinically acceptable bias of 2.1 % which is comparable to other studies [25,[27], [28], [29]]. Everolimus, however, demonstrated positive proportional bias with a slope of 1.35 on regression analysis, as well as a large positive bias of 41.2 %. Similar proportional biases for everolimus were reported by Lee et al. (slope 1.22) and Verstraete et al. (slope 1.21) using the Roche ECLIA assay [31,32]. Furthermore, Shipkova et al. reported a bias of 34.2 % between the Roche Everolimus ECLIA method and LC-MS/MS [30], while Verstraete et al. reported a bias of 26.5 %–36.5 % [31]. For sirolimus by ECLIA, we observed a median positive bias of +2.22 μg/L, which echoed the findings of Lee et al. who reported a mean bias of +2 μg/L for the Roche ECLIA method compared to LC-MS/MS in an Asian population [32]. In contrast, Stove et al. reported a mean bias of 0.41 μg/L compared to LC-MS/MS in a study performed in a European population [34]. Our findings are consistent with published literature and highlight the importance of performing method comparison studies across population groups, given the impact of pharmacogenomics on drug metabolite formation.

Bias has important clinical implications, especially when a single therapeutic target is used, regardless of the measurement methodology. For method comparisons, the International Association for Therapeutic Drug Monitoring and Clinical Toxicology (IATDMCT) recommends a slope of < ±10 % and a linear regression intercept that does not differ appreciably from zero compared to the reference method. An allowable bias of <5.8 % is also discussed in the context of a hypothetical total allowable error (TEa) of 15 % [15]. In our study, the bias for both everolimus and sirolimus exceeded two of these parameters. Moreover, the positive bias observed with the ECLIA method for everolimus and sirolimus resulted in a clinical misclassification rate of 27.9 % and 19.2 %, respectively, when compared to LC-MS/MS. Such misclassifications, which affect the distribution between sub-therapeutic, therapeutic and supra-therapeutic concentrations, pose risks for transplant patients, potentially leading to incorrect dose adjustments [10,11]. As a result, a patient's dose may be dependent on whether they are being monitoring with an LC-MS/MS or ECLIA method for everolimus and sirolimus. This carries significant clinical implications and warrants further investigation, including potential outcome studies going forward. Guidance on the adoption or establishment of method-specific therapeutic ranges, which is currently not provided by IATDMCT and IFCC guidelines [14,15], may be necessary and should be clearly outlined.

There are various explanations for the observed bias. There is currently no certified reference material or reference method for cyclosporine, everolimus and sirolimus, which limits calibrator traceability, resulting in possible calibration bias [15]. Secondly, cross-reactivity from drug metabolites is of particular importance for the measurement of these drugs [18]. The Roche ECLIA for everolimus has a reported cross-reactivity of 21.3 % for 24-hydroxy everolimus, 15.4 % for 25-hydroxy everolimus and 109.3 % for 40-phosphatidylcholine everolimus [44]. For sirolimus, the reported cross reactivity of 12-hydroxy sirolimus, 27-O-Desmethyl sirolimus and 39-O-Desmethyl sirolimus is 10.4 %, 65.0 % and 108.6 %, respectively [44]. Importantly, the immunosuppressive activity of these metabolites for both drugs is not yet fully elucidated, but is reported to be less than the parent drugs [15,18]. Cyclosporine metabolism yields many metabolites (AM1, AM9, AM4N, AM19, AM1c) of varying immunosuppressive activity [14,18]. The Roche ECLIA method for cyclosporine reports non-detectable cross-reactivity from AM19, AM1c and AM1c9, 2 % cross-reactivity for AM1 and AM4N and 6 % cross-reactivity for AM9 [44]. The efforts to decrease cross-reactivity in newer immunoassays are evident in our results for cyclosporine.

Imprecision needs to be considered along with bias for any measurement procedure. The calculated imprecision of the assay is acceptable according to IATDMCT analytical performance specifications, which recommend a minimum CVa of <10 % for immunoassay-based methods [15]. Our imprecision estimates for the Roche ECLIA method are comparable to CVa estimates in other studies [[23], [24], [25],[27], [28], [29], [30], [31], [32], [33], [34], [35]]. Another component of precision which should be considered is the variability among different clinical samples. The wide scatter of biases in our difference plots can partially be explained by heterogeneity among individuals [43] and the resulting variation of ISD metabolites [44]. This heterogeneity or imprecision between individuals means that a consistent level of bias is difficult to specify in our population.

Lastly, the practical aspects of these methods must be considered. Laboratories in resource-limited settings lack widespread access to LC-MS/MS [50], necessitating the use of more accessible methods like ECLIA. Both ECLIA and LC-MS/MS for ISD measurement require manual sample preparation, including cell lysis and extraction, increasing the workload compared to general automated immunoassay analysis. However, ECLIA is more convenient with fewer pre-treatment steps and less stringent staff requirements than LC-MS/MS making it better suited for resource-limited laboratories, such as those in sub-Saharan Africa.

This study has several strengths. It is novel in that it is the first method comparison study for ISD to be conducted in an African population. Secondly, remnant patient samples, which allow for a better assessment of the effect of variable metabolites, were used in this study as opposed to spiked samples. Furthermore, analysis of samples by ECLIA was conducted within 2 h of LC-MS/MS analysis for all samples and there were duplicate LC-MS/MS measurements for all samples. Finally, this study has a few limitations. Most samples for sirolimus and everolimus were within the therapeutic range with very few samples in the toxic range which limited assessment of correlation and concentration-dependent bias at higher concentrations. The concentrations assessed in our study do, however, represent those commonly encountered in clinical practice. Secondly, the study made use of anonymised remnant samples and therefore stratification of results into different transplant types (a recommendation by the IATDMCT) as well as consideration of factors such as time after transplantation, differences in therapeutic regimens and differences in ethnicity was not possible and would have resulted in underpowered findings. The tacrolimus immunoassay was not assessed in this study due to financial constraints. Acceptable analytical performance in the African population cannot be assumed and should be assessed in future studies.

In conclusion, this study, the first to be conducted in an African population, demonstrated significant bias between ECLIA and LC-MS/MS for everolimus and sirolimus measurement. In contrast, the cyclosporine immunoassay demonstrated comparability with LC-MS/MS in this patient population. The lack of comparability between these methods for everolimus and sirolimus measurement, highlighted by this study and other literature, is noteworthy. Although not unique to ISDs, this problem is perhaps under-recognised in the transplant community and the clinical consequence of these differences requires further investigation and possibly patient outcome studies to explore the potential risks posed by this non-comparability.

## CRediT authorship contribution statement

**Amy Strydom:** Writing – review & editing, Writing – original draft, Project administration, Methodology, Funding acquisition, Formal analysis, Data curation, Conceptualization, Visualization. **Doreen Jacob:** Writing – review & editing, Supervision, Methodology. **Taryn Pillay:** Writing – review & editing, Supervision. **Refeletse Malahlela:** Project administration. **Sean Currin:** Writing – review & editing, Supervision, Conceptualization, Project administration, Funding acquisition, Methodology.

## Ethical approval

The study was approved by the Human Research Ethics Committee of the University of the Witwatersrand (Clearance number: M230642).

## Research funding

Funding for LC-MS/MS analysis of patient samples was provided by NHLS K-Project Funding (PR2346632). Roche Diagnostics supplied the ECLIA reagent kits and the other consumables required for analysis by ECLIA.

## Declaration of competing interest

The authors declare the following financial interests/personal relationships which may be considered as potential competing interests:

The authors report that financial support was provided by the 10.13039/501100010753National Health Laboratory Service and 10.13039/100016545Roche Diagnostics. The authors declare that they have no known competing financial interests or personal relationships that could have appeared to influence the work reported on this paper.

## Data Availability

Data will be made available on request.
